# Epidemiological characteristics and risk factors of lung adenocarcinoma: A retrospective observational study from North China

**DOI:** 10.3389/fonc.2022.892571

**Published:** 2022-08-05

**Authors:** Daojuan Li, Jin Shi, Xiaoping Dong, Di Liang, Jing Jin, Yutong He

**Affiliations:** Cancer Institute, The Fourth Hospital of Hebei Medical University, Shijiazhuang, China

**Keywords:** lung adenocarcinoma, epidemiology, risk factors, never-smokers, North China

## Abstract

**Background:**

The main aim of the study was to determine the risk factors of lung adenocarcinoma and to analyze the variations in the incidence of lung adenocarcinoma according to time, sex, and smoking status in North China.

**Methods:**

Patients with lung cancer in local household registries diagnosed and treated for the first time in the investigating hospital were enrolled from 11 cities in North China between 2010 and 2017. Baseline characteristics and tumor-related information were extracted from the patients’ hospital medical record, clinical course records, and clinical examination. Some of the variables, such as smoking, alcohol consumption, medical history, and family history of cancer, were obtained from interviews with the enrolled patients. The statistical method used were the chi-square test and multi-factor logistic regression analysis. The time trend was statistically analyzed using Joinpoint regression models, and *p* values were calculated.

**Results:**

A total of 23,674 lung cancer cases were enrolled. People in severely polluted cities were at higher risk for lung adenocarcinoma (*p* < 0.001). Most patients with lung adenocarcinoma had no history of lung-related diseases (*p* = 0.001). Anatomically, lung adenocarcinoma was more likely to occur in the right lung (*p* < 0.001). Non-manual labor workers were more likely to develop from lung adenocarcinoma than manual workers (*p* = 0.015). Notably, non-smokers were more likely to develop lung adenocarcinoma than smokers (*p* < 0.001). The proportion of lung adenocarcinoma increased significantly in Hebei Province (*p* < 0.001). Among non-smokers, the proportion of lung adenocarcinoma showed a higher rise than in smokers (*p* < 0.001).

**Conclusions:**

Lung adenocarcinoma is the most common histological type of lung cancer in North China (Hebei Province), and the proportion of lung adenocarcinoma is increasing, especially among non-smokers. Lung adenocarcinoma is more common in women, severely polluted cities, individuals with no history of lung-related diseases, in the right lung, and in non-smokers. These can serve as a great guide in determining the accuracy of lung adenocarcinoma high-risk groups and lung cancer risk assessment models.

## Background

According to GLOBOCAN 2018, there were 2.09 million cases of lung cancer worldwide in 2018, accounting for 11.6% of all cancer incidences, and approximately 1.76 million lung cancer deaths, accounting for 18.4% of all cancer deaths, and lung cancer ranked first among all malignant tumor incidences and deaths. Moreover, there were 1.225 million new cases of lung cancer in Asia with 1.069 million lung cancer deaths, accounting for 58.5% of the incidence and 60.7% of the mortality of lung cancer worldwide (1). In China, lung cancer was the leading cancer in men and the second leading cancer in women with 550,000 and 278,000 new lung cancer cases, respectively. Lung cancer was the leading cancer in both men and women with 455,000 and 202,000 new lung cancer cases, respectively (2). Approximately 85% of lung cancers were non-small cell lung cancers, and 15% were small cell lung cancers. The following histological subtypes of non-small cell lung cancer were distinguished: adenocarcinoma, accounting for 38.5% of all lung cancers, and squamous cell carcinoma, accounting for 20% (3). A lung cancer study in the United States showed that the incidence of adenocarcinoma in men and women from 2004 to 2009 was 24.5/100,000 and 20.0/100,000, the incidence of squamous cell carcinoma was 18.8/100,000 and 8.5/100,000, and the incidence of small cell lung cancer was 9.8/100,000 and 7.9/100,000, respectively (4). Lung cancer was also the leading cause of cancer incidence and mortality in North China (Hebei Province) (5). Lung adenocarcinoma, the most common histological type of non-small cell lung cancer, has been on the rise in most countries over the past few decades (6), with a 5-year relative survival of only 12.8% (7). The increasing incidence of lung adenocarcinoma has been a subject of global interest. However, there is currently no report on the risk factors and epidemiological characteristics of lung adenocarcinoma in North China (Hebei Province). Therefore, this study aimed to determine the risk factors of lung adenocarcinoma and analyze the variations in the incidence of lung adenocarcinoma according to time, sex, and smoking status based on the distribution of lung adenocarcinoma in Hebei Province.

## Methods

### Study design and participants

Lung cancer clinical diagnosis and treatment information was collected from 133 hospitals in 11 cities of Hebei Province from 2010 to 2017: Shijiazhuang City, Baoding City, Tangshan City, Handan City, Xingtai City, Cangzhou City, Hengshui City, Langfang City, Qinhuangdao City, Chengde City, and Zhangjiakou City. Among the cities, Qinhuangdao City, Chengde City, and Zhangjiakou City were defined as the lightly polluted cities, while the other cities were severely polluted cities (8). Relevant variables were extracted from the patients’ hospital medical records, clinical course records, clinical examination, and clinical imaging studies. Baseline characteristics included the following: sex, age at diagnosis, marital status, occupation, height and weight at admission, blood type, smoking history, alcohol consumption, history of lung-related diseases, and family history of cancer. Tumor-related information was as follows: pathological type, diagnosis basis, grade, and sub-site of lung cancer patients. The inclusion criteria of study participants were as follows (1): admitted to hospital from 1 January 2010 to 31 December 2017; (2) new cases that were first diagnosed or treated in the investigating hospital and had not undergone surgery, radiotherapy, or chemotherapy in the previous hospital; and (3) cases of local household registration. The exclusion criterion included multiple primary or metastatic cancer cases.

Some of the variables, such as smoking, alcohol consumption, medical history, and family history of cancer, were obtained by interviewing the enrolled patients. Other variables were obtained by extracting information from the patients’ medical records.

The occupation classification in the questionnaire included non-manual labor and manual labor. Non-manual labor included persons in charge of party organs, mass organizations, social organizations, enterprises, and institutions; professional and technical personnel; and office and related personnel. Manual labor workers included social production service and life service personnel; agriculture, forestry, animal husbandry and fishery production, and auxiliary personnel; production and related personnel; soldiers; and other personnel who cannot be classified.

Lung cancer was mainly diagnosed by the following methods. Lung cancers were diagnosed by primary histology; that is, the pathological type of lung cancer was diagnosed by surgery or outpatient biopsy. Cytology and blood sample diagnosis referred to the diagnosis of lung cancer by sputum exfoliated cytology. Biochemical and immunological diagnosis referred to the diagnosis of lung cancer by biochemical, immunological, and tumor marker tests. Clinical diagnosis referred to the diagnosis of lung cancer by collecting medical history data through the patients’ clinical symptoms and signs and by asking patients about their subjective symptoms. Secondary histology referred to the examination and diagnosis of patients with discomfort caused by a secondary metastatic cancer and tracking their primary cancer as lung cancer. Related disease history included pulmonary tuberculosis, chronic bronchitis, emphysema, asthma, silicosis/pneumoconiosis, others, and unknown.

Patient’s height and weight were from the medical record system. Body mass index (BMI) was calculated with the formula of BMI = weight (kg) ÷ height (m^2^). BMI was categorized into <18.5 kg/m^2^ (underweight), 18.5–24.9 kg/m^2^ (normal weight), and 25–29.9 kg/m^2^ (overweight) (9).

When comparing lung adenocarcinoma with other pathological types, cases with unknown information on relevant variables were excluded from the study.

### Data collection and quality control

An expert group including experts in various fields such as clinical medicine, epidemiology, health statistics, cancer registration, and specialized persons who had long been engaged in clinical data collection was established. An expert seminar was held to present the study design and conduct technical guidance and quality control, followed by the training meeting. Investigators were selected by the project site according to the workload, and all the project team underwent the same technical training. All investigators followed the standardized operating procedures strictly. Experts from the project team visited the project sites at least once a year, and the supervision included (1) supervision of the collection of lung cancer clinical diagnosis data, (2) data storage check, (3) checking whether the collected data met the inclusion and exclusion criteria, and (4) checking data completeness and extracting 5% of the newly collected data in the current year to verify the information by querying the original data again.

### Statistical analysis

The statistical methods used were the chi-square test and multi-factor logistic regression analysis. According to well-known statistical methods, univariate analysis was performed for factor analysis. Through univariate analysis, a variable with statistical significance was screened out. Univariate analysis was performed on the variables collected in the questionnaire of this study, including sex, age, smoking status, alcohol intake, occupation, related disease history, family history of cancer, BMI, areas of residence, marriage, blood type, position, and morphology. After univariate analysis, we selected statistically significant variables for multivariate analysis, including sex, age, smoking status, areas, related disease history, occupation, and position. The time trend was statistically analyzed using Joinpoint regression models, and *p*-values were calculated. All statistical analysis and data quality control were performed using the Statistical Package for Social Science (SPSS) (version 20, SPSS Inc., Chicago, IL, USA), SAS software (Version 9.3), and the Joinpoint software (version 4.8.0.1; National Cancer Institute, Rockville, MD, US). *p* ≤ 0.05, established on two-sided probabilities, was considered statistically significant.

## Results

### Distribution of characteristics in patients with lung cancer in Hebei Province, 2010–2017

This study enrolled 23,674 patients diagnosed with lung cancer for the first time in 11 cities of Hebei Province. Among the lung cancer patients, the male-to-female ratio was 1.94:1. The average age of all lung cancer patients was 68.91 ± 11.21 years old, and the largest number of lung cancer cases was observed in the 65–69 age group.

Among all the lung cancer cases, regarding the smoking situation, the number of non-smokers was 10,913 (46.2%), current smokers was 7,533 (31.8%), former smokers (those who had quit smoking) was 2,732 (11.5%), and the number of patients with an unknown smoking status was 2,496 (10.5%). Moreover, 14,976 patients (63.3%) never drank, 5,881 patients (24.8%) drank frequently, and the drinking status of 2,817 (11.9%) patients was unknown. There were 2,850 non-manual labor (12.0%) and 9,724 (41.1%) manual workers. Among the lung cancer cases, 19,357 (81.8%) had no history of related diseases, 1,643 (6.9%) had a history of related diseases, and 2,674 had unknown information. Moreover, there were 18,378 (77.7%) patients without family history of cancer, 1,735 (7.3%) with family history of cancer, and 3,561 with unknown family history of cancer. Furthermore, 727 (3.1%) patients had a BMI index < 18.5 kg/m^2^, 6,444 (27.2%) had a BMI between 18.5 and 24.9 kg/m^2^, and 3,135 (13.2%) had a BMI ≥ 25 kg/m^2^. Among the lung cancer patients, 17.8% lived in areas with light air pollution, while 82.2% lived in areas with heavy air pollution. Regarding marital status, the married proportion was the highest at 82.7%, and the other proportions were all lower than 15%. Regarding blood type, blood type A accounted for 6.3%, blood type B accounted for 8.4%, blood type O accounted for 7.1%, and blood type AB accounted for 2.9%, and the proportion of unknown blood type was high (75.3%). Regarding the distribution of lung subsites, 1.2% occurred in both lungs, 38.9% occurred in the left lung, and 50.5% occurred in the right lung. There were 8,121 cases (34.3%) of adenocarcinoma, 3,443 cases (14.5%) of squamous cell carcinoma, 2,843 cases (12.1%) of small cell carcinoma, 956 cases (4.0%) of other cancer types, and 8,311 cases (35.1%) of unknown type with no pathology. Regarding the diagnosis, 13,785 lung cancer cases (58.2%) were diagnosed by primary histology, 892 cases (3.8%) were diagnosed by cytology and blood samples, 642 cases (2.7%) were diagnosed by biochemistry and immunology, and 1,822 cases (7.7%) were clinically diagnosed. Moreover, 215 cases (0.9%) were diagnosed by secondary histology, 5,198 cases (22.0%) were diagnosed by other special examinations (x-ray, ultrasound, CT, MRI, endoscope, etc.), and 1,120 cases (4.7%) had an unknown diagnosis (**Table 1**).

**Table 1:** Distribution of clinical characteristics in patients with lung cancer in Hebei Province, 2010–2017.

Factor		Cases	%
Sex	Male	15,611	65.9
	Female	8,063	34.1
Age	<45	396	1.7
	45–64	7,639	32.2
	≥65	15,639	66.1
Smoking status	Never	10,913	46.2
	Current	7,533	31.8
	Former	2,732	11.5
	Unknown	2,496	10.5
Alcohol intake	Never	14,976	63.3
	Current	5,881	24.8
	Unknown	2,817	11.9
Occupation	Non-manual labor	2,850	12.0
	Manual labor	9,724	41.1
	Unknown	11,100	46.9
Related disease history	No	19,357	81.8
	Yes	1,643	6.9
	Unknown	2,674	11.3
Family history of cancer	No	18,378	77.7
	Yes	1,735	7.3
	Unknown	3,561	15.0
BMI	<18.5	727	3.1
	18.5–24.9	6,444	27.2
	≥25	3,135	13.2
	Unknown	13,368	56.5
Areas	Light pollution	4,222	17.8
	Severe pollution	19,452	82.2
Marriage	Unmarried	295	1.2
	Married	19,568	82.7
	Widowed	536	2.3
	Divorced	199	0.8
	Unknown	3,076	13.0
Blood type	A	1,494	6.3
	B	1,999	8.4
	O	1,676	7.1
	AB	681	2.9
	Unknown	17,824	75.3
	Whole lung	301	1.2
Position	Left lung	9,205	38.9
	Right lung	11,947	50.5
	Unknown	2,221	9.4
	Adenocarcinoma	8,121	34.3
Morphology	Small cell carcinoma	2,843	12.1
	Squamous cell carcinoma	3,443	14.5
	Others	956	4.0
	Unknown (no pathology)	8,311	35.1

**Figure 1:**
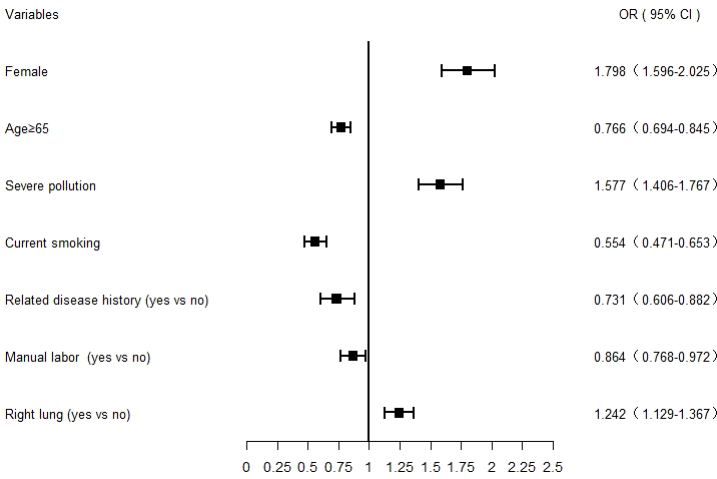
Forest plot of lung adenocarcinoma risk factors analyzed using a multivariate logistic regression model. The squares and bars represent the odds ratio (OR) with a 95% confidence interval (CI).

Lung adenocarcinoma was compared with other pathological types after excluding variables with unknown information. Through univariate analysis, variables with *t* statistical significance were obtained. Variables such as sex, age, smoking status, alcohol intake situation, occupation, related disease history, family history of cancer, BMI, air pollution level in areas, and subsites were included in the multivariate logistic regression analysis model. The multivariate regression findings suggested that women were at higher risk for lung adenocarcinoma than men (*p* < 0.001). Individuals over 65 years old had a lower risk of developing lung adenocarcinoma than those younger than 65 years old (*p* < 0.001). Notably, non-smokers were more likely to develop lung adenocarcinoma than smokers (*p* < 0.001). Non-manual labor workers were more likely to develop lung adenocarcinoma than manual workers (*p* = 0.015). The risk of lung adenocarcinoma was higher for people without a history of lung-related diseases (*p* = 0.001). Compared with people living in lightly polluted cities, those living in severely polluted cities were at higher risk for lung adenocarcinoma (*p* < 0.001). Moreover, lung adenocarcinoma occurred more frequently in the right lung than in the whole lung (*p* < 0.001) (**Table 2** and **Figure 1**).

**Table 2:** Distribution of clinical characteristics in patients with lung adenocarcinoma (%) in Hebei Province, 2010–2017.

Characteristic	Adenocarcinoma	Others	*X* ^2^	*p*
Sex				
Male	4,641 (57.1)	5,502 (76.0)	604.774	<0.001^*^
Female	3,480 (42.9)	1,740 (24.0)		
Age				
0–44	192 (2.4)	119 (1.6)	80.504	<0.001^*^
45–64	3,207 (39.5)	2,409 (33.3)		
≥ 65	4,722 (58.1)	4,714 (65.1)		
Smoking status				
Never	4,604 (60.2)	2,688 (39.5)	623.968	<0.001^*^
Current	2,259 (30.0)	2,965 (43.6)		
Former	752 (9.8)	1,149 (16.9)		
Alcohol intake				
Never	5,649 (74.8)	4,391 (65.9)	134.88	<0.001^*^
Current	1,902 (25.2)	2,270 (34.1)		
Occupation				
Non-manual labor	902 (21.9)	802 (19.1)	10.07	0.002^*^
Manual labor	3,218 (78.1)	3,473 (80.9)		
Related disease history				
No	7,137 (94.6)	6,114 (91.3)	62.595	<0.001^*^
Yes	403 (5.3)	584 (8.7)		
Family history of cancer				
No	6,700 (90.9)	5,804 (90.8)	0.029	0.866
Yes	672 (9.1)	588 (9.2)		
BMI				
<18.5	204 (5.8)	223 (5.8)	45	0.461
18.5–24.9	2,162 (61.4)	2,416 (62.7)		
≥25	1,157 (32.8)	1,214 (31.5)		
Areas				
Light pollution	1,120 (13.8)	1,645 (22.7)	206.538	<0.001^*^
Severe pollution	7,001 (86.2)	5,597 (77.3)		
Position				
Whole lung	97 (1.3)	89 (1.3)	16.199	<0.001^*^
Left lung	3,191 (41.3)	3,088 (44.6)		
Right lung	4,430 (57.4)	3,745 (54.1)		

* P<0.01, was considered statistically significant.

### Distribution changes of different histological types of lung cancer

**Figure 2:**
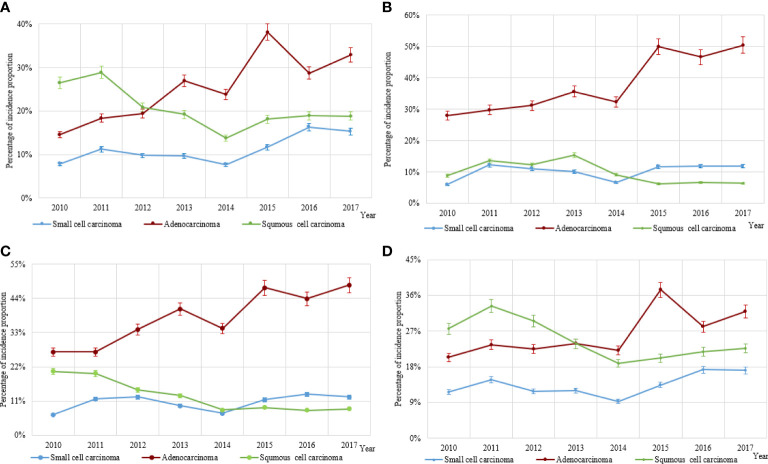
The incidence distribution of the three main histological types of lung cancer (lung adenocarcinoma, squamous cell carcinoma, and small cell carcinoma). **(A)** The incidence distribution of the three main histological types of lung cancer in men. **(B)** The incidence distribution of three main histological types of lung cancer in women. **(C)** The incidence distribution of three main histological types of lung cancer in patients with never-smoking lung cancer. **(D)** The incidence distribution of three main histological types of lung cancer in patients with smoking lung cancer.

The incidence trends of lung cancer in Hebei Province from 2010 to 2017 were analyzed by histological type. The proportion of lung adenocarcinoma (38.90%) in 2017 was 1.98 times higher than in 2010 (19.60%) (*p* < 0.001). However, the changes in the proportion of small cell carcinoma and squamous cell carcinoma were not statistically significant in both sexes (*p* = 0.100). For male patients, the proportions of lung adenocarcinoma and small cell carcinoma increased by 93.67% and 125.34%, respectively, in 2017 compared to 2010 (*p* < 0.001 and *p* < 0.001), whereas the proportion of lung squamous cell carcinoma decreased by 29.06% from 2010 to 2017 (*p* < 0.001) (**Figure 2A**). For female patients, between 2010 and 2017, the proportion of lung adenocarcinoma increased by 80.36% (*p* < 0.001), and the proportion of lung squamous cell carcinoma decreased by 28.41% (*p* < 0.001) (**Figure 2B**).

The histological types of lung cancer were analyzed by stratifying according to smoking status. The study suggested that there was a rise in the proportion of lung adenocarcinoma of 80.22% in never-smokers during 2010–2017 (*p* < 0.001). Moreover, the change in the proportion of small cell carcinoma was not statistically significant (*p* = 0.200), and there was a decline in the proportion of lung squamous cell carcinoma of 58.54% (*p* < 0.001) (**Figure 2C**). Among smokers, the proportion of lung adenocarcinoma similarly increased by 56.37% from 2010 to 2017 (*p* < 0.001), while the proportion of squamous cell carcinoma decreased by 17.75% (*p* < 0.001) (**Figure 2D**).

## Discussion

Lung cancer is the leading cause of cancer morbidity and mortality, accounting for one-fifth of all cancer deaths worldwide (1). Over the past two decades, the incidence of lung adenocarcinoma has been on the rise worldwide, and lung adenocarcinoma has become the most common lung cancer subtype (10). Investigating the risk factors of lung adenocarcinoma and analyzing how the incidence of lung adenocarcinoma varies according to time, sex, and smoking status are particularly important. This study focused on the analysis of the risk factors and epidemiological trends of lung adenocarcinoma in North China (Hebei Province) to provide an important reference for the screening and precise prevention of lung adenocarcinoma in high-risk groups.

Overall, the proportion of adenocarcinoma, squamous cell carcinoma, and small cell lung carcinomas in Hebei Province was consistent with that in the United States, Korea, and Australia (11–13). The risk factors of lung adenocarcinoma, the most pathological type of lung cancer in Hebei Province, was analyzed. The multivariate regression analysis findings revealed that lung adenocarcinoma was more common in women, individuals younger than 65 years old, those living in severely polluted cities, non-smokers, those with no history of lung-related diseases, non-manual labor workers, and the right lung. Many lung cancer studies worldwide found that among lung cancer cases in Canada, Denmark, Germany, New Zealand, the Netherlands, the United States, (Lima) Peru, and Southern Sweden, adenocarcinoma was more common in women and in younger patients (14–16).

A study from nine European countries manifested that the risk of lung cancer increased by 18% with PM2.5 increasing every 5 mg/m^3^, which may be due to lung adenocarcinoma (HR: 1.55, 95% CI: 1.05–2.29) (17, 18). In Canada, researchers found that a 0.01 mg/m^3^ increase in PM2.5 was inversely related to lung cancer (HR: 1.34; 95% CI: 1.10–1.65), with the strongest associations with adenocarcinoma (HR: 1.44; 95% CI: 1.06–1.97) (19). One study in the US indicated that for every 0.01 mg/m^3^ increase in PM2.5 level, the hazard rate of lung adenocarcinoma (HR: 1.31, 95% CI: 0.87–1.97) also increased (20). Moreover, in one meta-analysis, lung adenocarcinoma was more strongly associated with PM2.5 and PM10 (RR per 10 μg/m^3^, 1.40 [95% CI: 1.07–1.83] and 1.29 [95% CI: 1.02–1.63]) compared with other pathological types (21). A study in Taiwan Province of China also concluded that changes in PM2.5 levels can affect the incidence of lung adenocarcinoma and patient survival (22). Air pollution and the PM2.5 concentration were severely high in Hebei Province (23–25). As in our study, they may increase the risk of lung adenocarcinoma.

Although ever smoking strongly correlated with increased risk for lung adenocarcinoma, lung adenocarcinoma was the most common subtype among never smokers (26, 27). In this study, the result found that non-smokers were more likely to develop lung adenocarcinoma than smokers. An earlier study in Singapore showed that approximately 70% of never-smoking lung cancer patients were diagnosed with adenocarcinoma (28). The World Trade Center Environmental Health Center conducted a study among people who were exposed and possibly inhaled dust and fumes from the destruction of the World Trade Center towers in 2001. The results showed that lung adenocarcinoma was more common in never-smokers compared to ever-smokers (72% *vs*. 65%) and more common in women compared to men (70% *vs*. 65%) (29). One study in China showed that though the proportion of lung adenocarcinoma increased in smokers and non-smokers, lung adenocarcinoma was more common in non-smokers (30). However, the result from a national health examination program in Korea was inconsistent with that of this study. The hazard ratios for lung adenocarcinoma were significantly higher in male current smoker than in never-smokers, while they were not different for female patients (31). Despite this, non-smoker lung cancer had been classified as an independent disease entity, which is different from smoker lung cancer. Gene sequencing technology has enabled us to truly understand the difference in lung adenocarcinoma between smokers and non-smokers at the microscopic molecular level. Most of the driver gene alterations were identified in lung adenocarcinoma in never-smokers. However, no such available molecular targets exist for smoker lung cancer (32, 33). This study found that non-smokers were more likely to develop lung adenocarcinoma, and because the mechanisms of lung adenocarcinoma in smokers and non-smokers were completely different, we should pay more attention to the screening of lung adenocarcinoma among non-smokers at high risk of lung cancer. It was of great significance to strengthen the screening of lung adenocarcinoma among non-smokers at high risk of lung cancer for related genes and to detect and diagnose lung adenocarcinoma early, improving the chances of surgery for patients and prolonging the survival time.

This study also found that most patients with lung adenocarcinoma had no history of lung-related diseases. Anatomically, lung adenocarcinoma was more likely to occur in the right lung. In addition, this study revealed that non-manual labor workers had a greater risk of lung adenocarcinoma compared with manual workers. These new findings will have a significant impact on the prevention of lung adenocarcinoma. We should strengthen the screening of lung adenocarcinoma for non-manual workers and people without a history of lung-related diseases to achieve early detection, early diagnosis, and early treatment. For designated lung adenocarcinoma high-risk groups, attention should be paid to the examination of the right lung. However, these findings may require more research to confirm.

Overall, the proportion of lung adenocarcinoma increased significantly in Hebei Province, but the proportion of lung squamous cell carcinoma decreased during 2010–2017. Among non-smokers, the proportion of lung adenocarcinoma showed a higher rise than in smokers. This implied that lung adenocarcinoma is still the main histological type of lung cancer.

This study showed that lung adenocarcinoma, one of the types of non−small cell lung carcinoma, was the most common form of lung cancer among non−smokers (34). In 10 years, lung cancer characteristics have changed: more women, more never-smokers, and more adenocarcinomas in France (35). In a study among Chinese women, the proportion of never-smokers with adenocarcinoma increased significantly compared with smokers (36). The prevention of lung adenocarcinoma has to be given more prominence, especially among non-smokers. The incidence of adenocarcinoma increased in the United States between 2006 and 2010, but there was a greater reduction in the rate of decline of squamous cell carcinoma than before (37). One study proved that the adenocarcinoma incidence trends were consistent with smoking trends; however, the relative risk with smoking for adenocarcinoma was lower than that for squamous cell carcinoma and small cell carcinoma (38). Our study showed that smoking had the greatest effect on squamous cell carcinomas, a lesser effect on lung adenocarcinomas (**Supplementary Tables 1, 2**), and no statistically significant effect on small cell carcinomas (**Supplementary Table 3**). In China, from 1990 to 2015, the age-standardized prevalence of daily smoking decreased significantly by 22.4% (95% UI: 20.7%–24.0%) and 48.4% (95% UI: 41.2%–55.1%) for men and women, respectively (39). Declining smoking rates in China may be one of the main reasons for this result.

In this study, we found that the incidence of lung adenocarcinoma was increasing, especially in non-smokers, revealing the changing trends in the pathological types of lung cancer. At present, few studies have reached this conclusion, which can have a great guiding effect on the selection of high-risk lung cancer groups for screening. In addition, we also found that lung adenocarcinoma was more common in severely polluted cities, non-smokers, individuals with no history of pulmonary diseases, non-manual labor workers, and the right lung. These conclusions have important implications. First, the conclusions have a key guiding significance for determining the accuracy of the lung cancer risk assessment model. In addition, because cancer screening programs do not currently cover the whole country, this can be more beneficial to high-risk populations through lung cancer screening, improve the detection rate, and reduce healthy individuals from receiving numerous unnecessary tests, especially those that can cause some pain and harm.

The present study also had some shortcomings. The study enrolled 23,674 lung cancer patients. For smoking status, the number of unknowns accounted for 10.5%. In the future, we need to pay attention to the integrity of variable information collection or choose an adequate method to supplement missing data; however, we currently only presented the results of real data. In this study, the main inclusion criterion for lung cancer patients was patients who were first diagnosed or treated in the hospital. Most patients with good economic conditions who were later found to have space occupancy by other means instead of a pathological diagnosis chose provincial or national specialized oncology hospitals to perform further examinations and make a pathological diagnosis. This led to the local hospital not being able to obtain the histological subtypes for the first diagnosis. Moreover, among the 35.1% of the total cases with unknown histological diagnosis, some of them may have had clear pathological types, but the local hospitals could not obtain them, which is also a limitation of this study.

## Conclusion

Lung adenocarcinoma is one of the most common histological types of lung cancer in North China (Hebei Province), and the incidence of lung adenocarcinoma is increasing, especially in non-smokers. Lung adenocarcinoma is more common in women, individuals younger than 65 years old, those living in severely polluted cities, non-smokers, those with no history of lung-related diseases, non-manual labor workers, and the right lung. These can play a great guiding role in determining the accuracy of lung adenocarcinoma diagnosis in high-risk groups and lung cancer risk assessment models.

## Data availability statement

The original contributions presented in the study are included in the article/**Supplementary Material**. Further inquiries can be directed to the corresponding author.

## Ethics statement

This study was reviewed and approved by the Ethics Committee of Hebei Medical University Fourth Hospital. Written informed consent to participate in this study was provided by the participants’ legal guardian/next of kin. Written informed consent to participate in this study was provided by the participants’ legal guardian/next of kin.

## Author contributions

DJL and YTH were involved in the design and data analysis of this study and drafted the manuscript. YTH was responsible for the oversight of the whole study and edited the manuscript. DJL conceived the review and edited the manuscript. DJL, JS, XPD, DL, and JJ collected and analyzed the data. All authors contributed to the article and approved the submitted version.

## Funding

This study was approved and supported by Department of Disease Control and Prevention, Hebei Provincial Health Commission Letter (2018) No. 5.

## Acknowledgments

We gratefully acknowledge the cooperation of all the population-based cancer registries in providing cancer statistics, data collection, sorting, verification, and database creation.

## Conflict of interest

The authors declare that the research was conducted in the absence of any commercial or financial relationships that could be construed as a potential conflict of interest.

## Publisher’s note

All claims expressed in this article are solely those of the authors and do not necessarily represent those of their affiliated organizations, or those of the publisher, the editors and the reviewers. Any product that may be evaluated in this article, or claim that may be made by its manufacturer, is not guaranteed or endorsed by the publisher.
