# Supplementary material for: Epidemiological characteristics and risk factors of lung adenocarcinoma: A retrospective observational study from North China
Source: Front Oncol. 2022 Aug 5;12:892571. doi: 10.3389/fonc.2022.892571 (PMC9389456; doi:10.3389/fonc.2022.892571)
Supplement: Supplementary file 2 [file Presentation_1.pptx]

## Slide 1
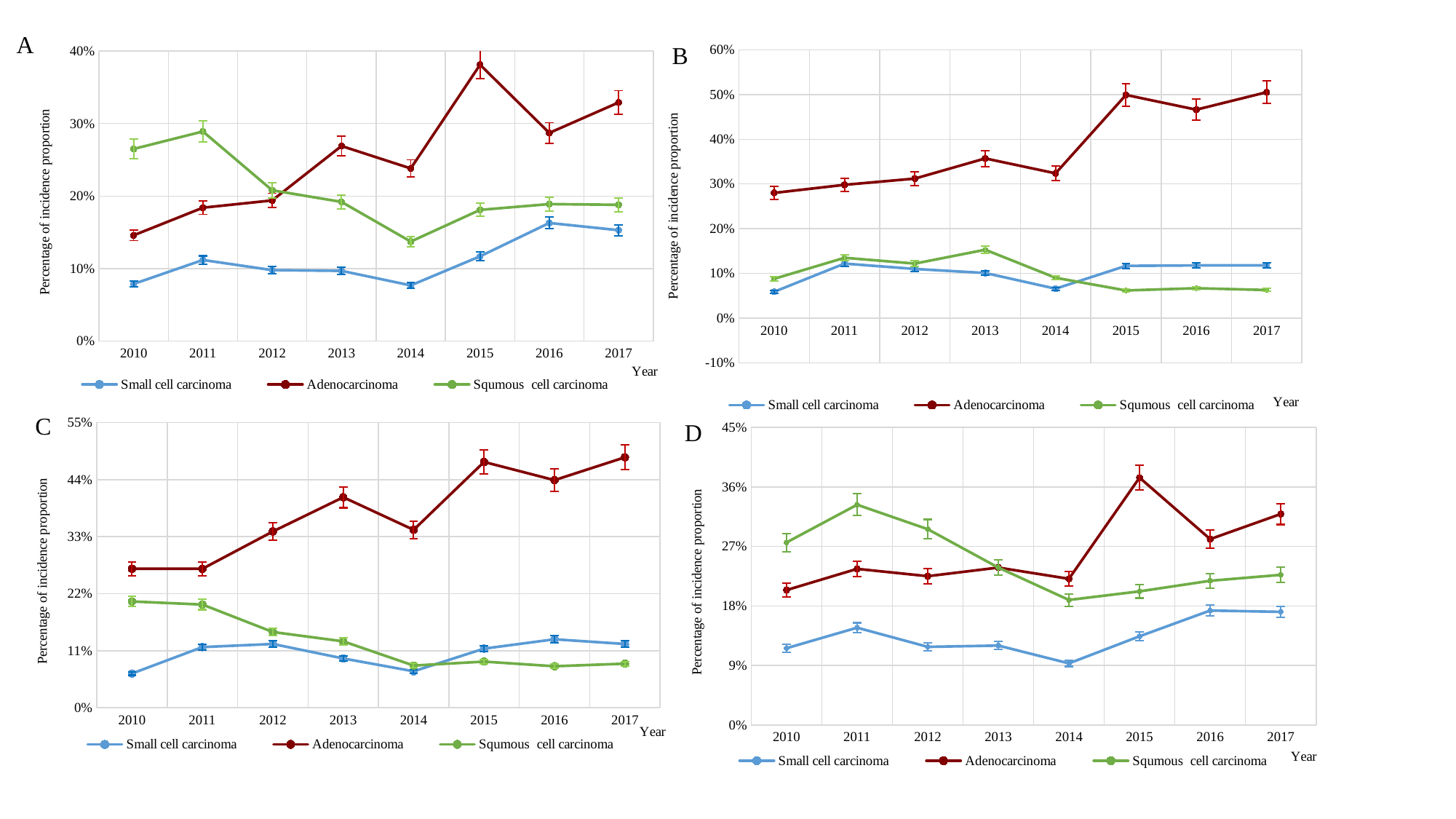

### Chart
| Category | Small cell carcinoma | Adenocarcinoma | Squmous cell carcinoma |
|---|---|---|---|
| 2010 | 0.079 | 0.146 | 0.265 |
| 2011 | 0.112 | 0.184 | 0.289 |
| 2012 | 0.098 | 0.194 | 0.208 |
| 2013 | 0.097 | 0.269 | 0.192 |
| 2014 | 0.077 | 0.23809086781999997 | 0.13733259999999997 |
| 2015 | 0.117 | 0.381 | 0.181 |
| 2016 | 0.163 | 0.287 | 0.189 |
| 2017 | 0.153 | 0.329 | 0.188 |
### Chart
| Category | Small cell carcinoma | Adenocarcinoma | Squmous cell carcinoma |
|---|---|---|---|
| 2010 | 0.059 | 0.28 | 0.088 |
| 2011 | 0.122 | 0.298 | 0.135 |
| 2012 | 0.11 | 0.312 | 0.122 |
| 2013 | 0.101 | 0.357 | 0.153 |
| 2014 | 0.0659237 | 0.32368777839999996 | 0.09038650999999999 |
| 2015 | 0.117 | 0.499 | 0.062 |
| 2016 | 0.118 | 0.466 | 0.067 |
| 2017 | 0.118 | 0.505 | 0.063 |
### Chart
| Category | Small cell carcinoma | Adenocarcinoma | Squmous cell carcinoma |
|---|---|---|---|
| 2010 | 0.066 | 0.268 | 0.205 |
| 2011 | 0.117 | 0.268 | 0.199 |
| 2012 | 0.123 | 0.34 | 0.146 |
| 2013 | 0.095 | 0.406 | 0.128 |
| 2014 | 0.07034489999999999 | 0.3431150399999999 | 0.08139959999999999 |
| 2015 | 0.114 | 0.474 | 0.089 |
| 2016 | 0.132 | 0.439 | 0.08 |
| 2017 | 0.123 | 0.483 | 0.085 |
### Chart
| Category | Small cell carcinoma | Adenocarcinoma | Squmous cell carcinoma |
|---|---|---|---|
| 2010 | 0.116 | 0.204 | 0.276 |
| 2011 | 0.147 | 0.236 | 0.333 |
| 2012 | 0.118 | 0.225 | 0.296 |
| 2013 | 0.12 | 0.238 | 0.238 |
| 2014 | 0.09305189999999999 | 0.221 | 0.18898740999999997 |
| 2015 | 0.134 | 0.374 | 0.202 |
| 2016 | 0.173 | 0.281 | 0.218 |
| 2017 | 0.171 | 0.319 | 0.227 |
