# Supplementary material for: Epidemiological characteristics and risk factors of lung adenocarcinoma: A retrospective observational study from North China
Source: Front Oncol. 2022 Aug 5;12:892571. doi: 10.3389/fonc.2022.892571 (PMC9389456; doi:10.3389/fonc.2022.892571)
Supplement: Supplementary file 3 [file Table_1.docx]

| supplementary Table 1 The multivariate logistic regression analysis with lung adenocarcinoma | | | | | | | | |
| --- | --- | --- | --- | --- | --- | --- | --- | --- |
| Factor |  | Coefficient | S.E | Waldc2 | *P* | OR | 95%CI |  |
| Gender | Female | 0.587 | 0.061 | 93.215 | 0.000 | 1.798 | 1.596～2.025 |  |
| Age | ≥65 | -0.267 | 0.050 | 28.487 | 0.000 | 0.766 | 0.694～0.845 |  |
| Areas | Severe pollution | 0.455 | 0.058 | 60.916 | 0.000 | 1.577 | 1.406～1.767 |  |
| Smoking status | Current | -0.590 | 0.083 | 50.100 | 0.000 | 0.554 | 0.471～0.653 |  |
| Related disease history | Yes | -0.313 | 0.096 | 10.728 | 0.001 | 0.731 | 0.606～0.882 |  |
| Occupation | Manual labor | -0.146 | 0.060 | 5.958 | 0.015 | 0.864 | 0.768～0.972 |  |
| Position | Right lung | 0.217 | 0.049 | 19.626 | 0.000 | 1.242 | 1.129～1.367 |  |

| supplementary table 2 Distribution of histology with lung cancer in Hebei Province, 2010-2017 | | | | | | | | | | | | |
| --- | --- | --- | --- | --- | --- | --- | --- | --- | --- | --- | --- | --- |
| Year | Male | | | Female | | | Never Smoking | | | Current Smoking | | |
|  | SCC | Ade | Sqcc | SCC | Ade | Sqcc | SCC | Ade | Sqcc | SCC | Ade | Sqcc |
| 2010 | 35 | 65 | 114 | 18 | 76 | 23 | 24 | 91 | 66 | 16 | 31 | 31 |
| 2011 | 55 | 101 | 138 | 32 | 92 | 42 | 40 | 114 | 74 | 29 | 38 | 58 |
| 2012 | 69 | 132 | 140 | 45 | 114 | 39 | 64 | 152 | 67 | 27 | 47 | 65 |
| 2013 | 131 | 348 | 238 | 66 | 245 | 105 | 85 | 360 | 111 | 66 | 127 | 131 |
| 2014 | 153 | 432 | 232 | 65 | 289 | 37 | 97 | 375 | 79 | 77 | 237 | 119 |
| 2015 | 450 | 1432 | 686 | 208 | 936 | 116 | 280 | 1207 | 224 | 259 | 738 | 377 |
| 2016 | 598 | 1046 | 694 | 222 | 864 | 123 | 330 | 1114 | 205 | 329 | 513 | 399 |
| 2017 | 497 | 1075 | 605 | 193 | 859 | 105 | 302 | 1180 | 199 | 285 | 555 | 384 |
| SCC, Small cell carcinoma; Ade, Adenocarcinoma; Sqcc, Squamous cell carcinoma | | | | | | | | | | | | |

| Supplementary table 3 The multivariate logistic regression analysis with lung subtype | | | | | | | | |
| --- | --- | --- | --- | --- | --- | --- | --- | --- |
| Factor |  | Reference | Coefficient | S.E | Waldc2 | *P* | OR | 95%CI |
| small cell lung cancer | | |  |  |  |  |  |  |
| Area | Severe pollution | Light pollution | -0.462 | 0.061 | 57.053 | 0.000 | 0.63 | 0.559～0.710 |
| Gender | Female | Male | -0.17 | 0.06 | 7.873 | 0.005 | 0.844 | 0.750～0.950 |
| Occupation | Manual labor | Mental work | 0.145 | 0.072 | 4.087 | 0.043 | 1.156 | 1.004～1.331 |
| squamous cell carcinoma | | |  |  |  |  |  |  |
| Age | ≥65 | 0～44 | 0.383 | 0.063 | 36.973 | 0.000 | 1.467 | 1.296～1.659 |
| Gender | Female | Male | -0.569 | 0.076 | 55.666 | 0.000 | 0.566 | 0.488～0.658 |
| Smoking status | Current | Never | 0.741 | 0.086 | 74.858 | 0.000 | 2.098 | 1.773～2.481 |
| Related disease history | Yes | No | 0.543 | 0.1 | 29.583 | 0.000 | 1.72 | 1.415～2.092 |
